# Rheumatoid Arthritis and Incidence of Twelve Initial Presentations of Cardiovascular Disease: A Population Record-Linkage Cohort Study in England

**DOI:** 10.1371/journal.pone.0151245

**Published:** 2016-03-15

**Authors:** Mar Pujades-Rodriguez, Bram Duyx, Sara L. Thomas, Dimitris Stogiannis, Anisur Rahman, Liam Smeeth, Harry Hemingway

**Affiliations:** 1 Farr Institute of Health Informatics Research, University College London, 222 Euston Road, London NW1 2DA, United Kingdom; 2 Leeds Institute of Biomedical and Clinical Sciences, MRC Medical Bioinformatics Centre, Worsley Building, University of Leeds, Leeds LS2 9JT, United Kingdom; 3 Department of Non-communicable Disease Epidemiology, London School of Hygiene and Tropical Medicine, Keppel Street, London WC1E 7HT, United Kingdom; 4 Centre of Rheumatology Research, Division of Medicine, Faculty of Medical Sciences, University College London, London WC1E 6JF, United Kingdom; Hunter College, UNITED STATES

## Abstract

**Introduction:**

While rheumatoid arthritis is an established risk factor for cardiovascular disease (CVD), our knowledge of how the pattern of risk varies for different cardiovascular phenotypes is incomplete. The association between rheumatoid arthritis and the initial presentation of 12 types of CVDs were examined in a contemporary population of men and women of a wide age range.

**Methods:**

CALIBER data, which links primary care, hospital and mortality data in England, was analysed. A cohort of people aged ≥18 years and without history of CVD was assembled and included all patients with prospectively recorded rheumatoid arthritis from January 1997, until March 2010, matched with up to ten people without rheumatoid arthritis by age, sex and general practice. The associations between rheumatoid arthritis and the initial presentation of 12 types of CVDs were estimated using multivariable random effects Poisson regression models.

**Results:**

The analysis included 12,120 individuals with rheumatoid arthritis and 121,191 comparators. Of these, 2,525 patients with and 18,146 without rheumatoid arthritis developed CVDs during a median of 4.2 years of follow-up. Patients with rheumatoid arthritis had higher rates of myocardial infarction (adjusted incidence ratio [IRR] = 1.43, 95%CI 1.21–1.70), unheralded coronary death (IRR = 1.60, 95%CI 1.18–2.18), heart failure (IRR = 1.61, 95%CI 1.43–1.83), cardiac arrest (HR = 2.26, 95%CI 1.69–3.02) and peripheral arterial disease (HR = 1.36, 95%CI 1.14–1.62); and lower rates of stable angina (HR = 0.83, 95%CI 0.73–0.95). There was no evidence of association with cerebrovascular diseases, abdominal aortic aneurysm or unstable angina, or of interactions with sex or age.

**Conclusions:**

The observed associations with some but not all types of CVDs inform both clinical practice and the selection of cardiovascular endpoints for trials and for the development of prognostic models for patients with rheumatoid arthritis.

## Introduction

Studies have reported a 1.5 to 2-fold increase in risk of acute myocardial infarction (AMI), cerebrovascular disease[1–5] or cardiovascular mortality[6–8] in people with rheumatoid arthritis (RA). The absolute cardiovascular risk in individuals with this chronic inflammatory condition has been estimated to be similar to that in non-RA individuals who are approximately ten years older[9], and research suggests that the risk is similar to that seen in people with type 2 diabetes[10–12]. Both traditional risk factors and underlying disease may contribute to the increased cardiovascular risk[13]. Possible mechanisms include chronic immune dysregulation and systemic inflammation[14,15], induction of cardiovascular risk factors by medication (e.g. some disease-modifying antirheumatic drugs [DMARDs], oral corticosteroids or non-steroidal anti-inflammatory drugs), or common risk factors for RA and cardiovascular disease (CVD) such as genes[16] or smoking[17,18].

Previous studies have included only a limited range of cardiovascular endpoints, generally AMI and all-cause stroke, and most have been small or were undertaken prior to the widespread use of modern therapies for RA and to the introduction of an earlier and more aggressive pharmacological management[19].

The availability of linked electronic health records from primary care, hospital, disease registries and mortality through CALIBER (CArdiovascular research using LInked Bespoke studies and Electronic health Records)[20–23], offers the opportunity to investigate the association between RA and the initial presentation of specific types of CVDs in a contemporary population in England. The validity of both risk factor and disease measurements in CALIBER is supported by findings replicating and extending anticipated associations between phenotypic definitions for the 12 most common symptomatic CVDs and smoking[21], systolic and diastolic blood pressure[23], diabetes[24], and socio-economic deprivation[22].

The objectives of this analysis were to compare the rates of specific types of CVDs in individuals with and without RA and no prior history of CVD; and to investigate whether associations differ according to disease duration, sex, age, or presence of other autoimmune diseases in a contemporary population.

## Methods

### Study Population

All patients with a recorded RA diagnosis among individuals registered in the general practices contributing data to CALIBER between January 1997 and March 2010 were included. For each of these, up to ten individuals matched on age (±5 years), sex and general practice were randomly selected amongst those not diagnosed with the disease on their matched inclusion date (concurrent sampling), who were alive and actively registered in the same practice (i.e. with a practice contact record within 1 year of the inclusion date to ensure that they had the same opportunity for clinical diagnosis and reporting of risk factors and outcomes). Inclusion criteria were age ≥18 years, no history of CVD and contributing at least one year of data after registering with the practice. Patients with juvenile RA, and those with no RA diagnosis but with prescribed DMARD or biologic therapy suggestive of RA disease were excluded from the non-RA group (Figure A in S1 File). Patient electronic medical records linked across four data sources were analysed: the Clinical Practice Research Datalink (CPRD)[25]; the Myocardial Ischaemia National Audit Project disease registry[26]; Hospital Episodes Statistics (HES); and the national death registry.

### Rheumatoid arthritis

Patients with RA were identified from electronic medical records in CPRD and HES using Read and International Classification of Disease (ICD) version 10 codes, respectively (Table A in S1 File). The validity of Read codes of RA has been demonstrated in studies conducted in this and similar medical databases[27,28]. Individuals with RA who additionally had supporting information for diagnosis (at least one diagnosis code for RA in both primary [CPRD] and secondary [HES] care, a DMARD or biologic therapy prescription [Table B in S1 File], or a visit to rheumatology clinic within 6 months of the diagnosis code; or a positive rheumatoid factor test or anti-cyclic citrullinated peptide antibody test after diagnosis), were included in primary analyses regardless of disease duration. Patients with no supporting information for diagnosis were also included in sensitivity analyses. For secondary analyses, individuals were classified into incident and prevalent RA cases, depending on whether diagnosis was made before or after the start of the observation period (‘index date’).

### Baseline covariates

Baseline covariates considered in the analysis were: sex, age, index of multiple deprivation[22], diagnosed diabetes mellitus[24], smoking status[21], body mass index (BMI), systolic blood pressure, medication use (≥2 prescriptions of nonsteroidal anti-inflammatory drugs, oral corticosteroids, blood pressure lowering drugs and statins), year of study start and other diagnosed autoimmune diseases (Table C in S1 File). The most recent measurement of quantitative variables such as blood pressure (or prescription for medication) recorded in CPRD up to one year before study entry was used to define baseline covariates. The index of multiple deprivation is a composite indicator that provides a relative ranking of areas across the United Kingdom according to seven domains of deprivation: i) income; ii) employment; iii) health and disability; iv) education, skills and training; v) barriers to housing and services; vi) crime; and vii) living environment[29]. Patients with coded diagnoses of diabetes (type 1, type 2 or uncertain type) recorded in CPRD or HES at or before study entry were considered to have diabetes at baseline. Definitions can be found at https://www.caliberresearch.org/portal/.

### Endpoints

The primary endpoints for the analysis were the initial presentation of fatal and non-fatal CVDs. CVD presentations studied were: i) cardiac diseases: stable angina, unstable angina, AMI, heart failure, cardiac arrest; ii) cerebrovascular diseases: transient ischaemic attack, ischaemic stroke, subarachnoid haemorrhage, intracerebral haemorrhage; and iii) peripheral vascular diseases: PAD, and abdominal aortic aneurysm. Secondary endpoints were two composite CVD outcomes defined as for the estimation of the 1998 and 2008 Framingham prediction scores, the coronary and CVD death composite (including stable angina, AMI, coronary heart diseases not otherwise specified, and any CVD death), and the fatal and non-fatal cardiovascular composite (additionally including non-fatal heart failure, transient ischaemic attack, ischaemic or haemorrhagic stroke, and PAD); and the first event of each CVD type (i.e. regardless of other earlier CVD presentations). Diagnosis codes used to define each endpoint can be found at http://www.caliberresearch.org/portal/.

### Study follow-up period

For individuals with RA the observation period began on the date when the patient fulfilled all the study inclusion criteria, or the date of first recorded diagnosis code for RA. For non-RA patients, the observation period started on the index date of the matched RA individual. The observation period for all patients ended on the date of cardiovascular endpoint diagnosis, death unrelated to the study endpoints, practice deregistration, last date of data collection in the practice, or diagnosis of RA (for patients who contributed follow-up to both the rheumatoid and non-rheumatoid disease groups).

### Statistical analysis

Incidence rates of events per 1000 person-years of follow-up with 95% confidence intervals were calculated in patients with and without RA, and separately for patients with prevalent or incident disease. To examine associations between disease and each study endpoint we used random-effects Poisson regression models accounting for clustering between practices. The strategy for adjustment was defined a priori. Models were initially adjusted for the confounding effects of age and sex, then additionally for established cardiovascular risk factors (multiple deprivation, diabetes mellitus, smoking status, BMI, and systolic blood pressure). Missing values of smoking status, systolic blood pressure, and BMI were multiply imputed (Multiple imputation in S1 File). Systolic blood pressure and BMI were incorporated into the models as continuous variables. Age as linear and quadratic terms. To explore possible pathways or mediators of any increase in risk, estimates were further adjusted for: i) lipid and blood pressure lowering medication, ii) diagnosis of another autoimmune disease, iii) anti-inflammatory drugs (nonsteroidal, and oral corticosteroid medication); and iv) calendar year of inclusion. Heterogeneity in associations across CVD endpoints was assessed with the I-squared[30], assuming independence in effects.

In secondary analyses, interactions with age and sex were investigated as well as associations with prevalent and incident RA. Furthermore, to assess the association between disease duration and CVD, we used time-dependent covariates. For this, we split the follow-up of individuals with incident RA into periods of disease duration (0–1, 2–4, 5–9 and ≥10 years) and only included individuals with prevalent RA who had their first recorded diagnosis 10 years or more before study entry (categorised in the ≥10 year duration period). Individuals with prevalent disease but shorter disease duration were excluded from this secondary analysis because of uncertainty about the time since diagnosis. In addition, because previous studies suggested that the risk of CVD is similar to that seen in people with type 2 diabetes[10–12], we compared estimates for the two diseases examining the overlap of confidence intervals.

Sensitivity analyses comprised: i) exclusion of individuals with another autoimmune disease; ii) restriction to individuals with at least 6 months of patient-time follow-up; and iii) exclusion of 2 years of follow-up prior to the diagnosis of RA for individuals who contributed to the disease and unexposed groups; iv) inclusion of individuals without supporting information for RA diagnosis (one data source); v) inclusion of first cardiovascular events irrespective of other earlier CVD presentations; and vi) comparison of estimates in the periods before and after the introduction of the pay for performance scheme in England (Quality of Outcomes Framework). This scheme financially rewards general practices for meeting a range of quality targets in several areas, including the improvement of management of chronic diseases and how practices are organised.

Analyses were performed using Stata v13.1 (StataCorp LP).

Approval was granted by the Independent Scientific Advisory Committee of the Medicines and Healthcare Products Regulatory Agency and the Myocardial Ischaemia National Audit Project Academic Group. The protocol was registered at clinicaltrials.gov (NCT02062021). Data was anonymised and de-identified prior to analysis.

## Results

### Patient characteristics

**Fig 1:**
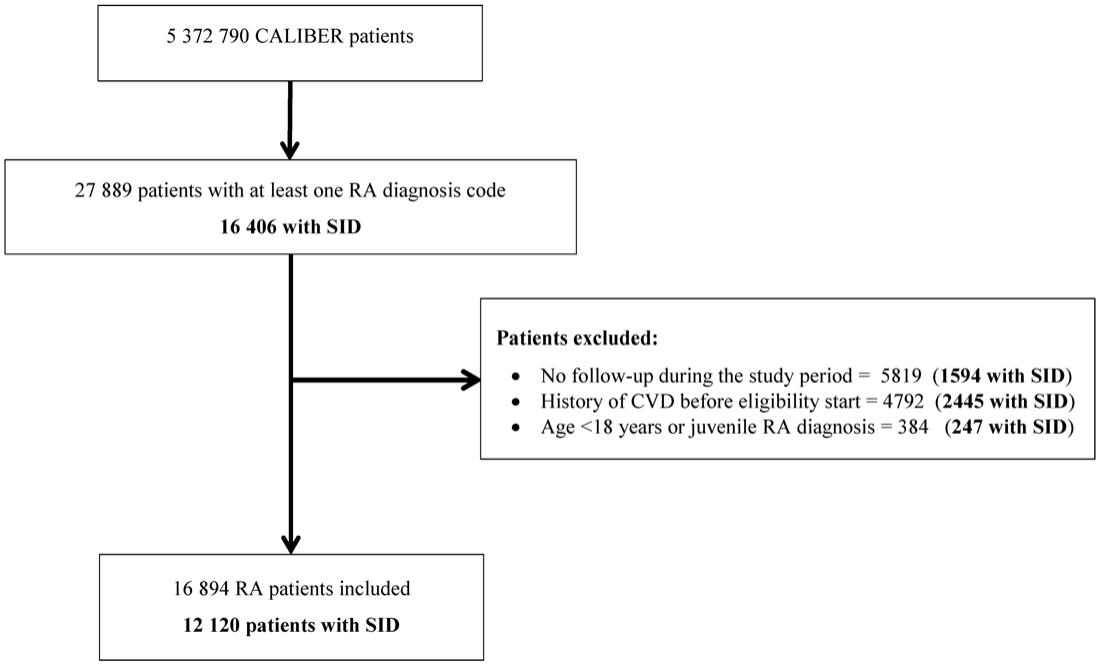
Study flow diagram. Note: CVD, cardiovascular disease; RA, rheumatoid arthritis; SID, supportive information for rheumatoid arthritis diagnosis.

In total 12,120 individuals with RA and supporting diagnostic information (Fig 1), and 121,191 matched non-RA individuals with no prior history of CVD were included in the primary analyses. Of these, 736 individuals contributed follow-up time to both the RA and non-RA groups. Fifty percent of individuals with RA (n = 6,095) had incident disease and 56.7% of those with prevalent disease had been diagnosed 5 years or more before study entry. Overall, median duration of registration in the practice was 10.7 years (IQR 4.5–20.4). Median age at study entry was 57 years [IQR 46–67] and 72.3% were women (Table 1). Individuals included in the study contributed 675,489 person-years of follow-up, with a median of 4.2 years. Of patients with RA, 54.0% received nonsteroidal anti-inflammatory drugs, 22.1% corticosteroids and 50.9% DMARDs or biologic therapy (69.0% of prevalent and 33.1% of incident rheumatoid disease patients) in the year before study entry. Eighty-three percent of individuals with RA had at least one recorded prescription of DMARDs or biologic therapy within 6 months of a diagnosis code. Diagnosis with another autoimmune disease was recorded in 16.1% of RA patients and 5.2% of non-RA patients. The distribution of traditional cardiovascular risk factors was similar among individuals with and without RA except for the higher prevalence of current or former smoking among patients with RA (41.9% vs. 33.4%). At baseline 3,835 (31.6%) patients with RA and 4,089 non-RA patients had results of rheumatoid factor testing recorded; a positive test was found in 45.8% and 6.5% of these patients, respectively.

**Table 1:** Characteristics* of individuals with and without rheumatoid arthritis.

	Patients without RA	All patients with RA
	N = 121,191	N = 12,120
**Sociodemographic factors**		
Age in years, median [IQR]	56 [45–67]	59 [48–68]
Women, n (%)	87,640 (72.3)	8764 (72.3)
Index of multiple deprivation in quintiles, n (%)		
1 (most deprived)	24,770 (20.5)	2329 (19.3)
5 (least deprived)	23,855 (19.8)	2627 (21.8)
Ethnicity, n (%)		
*White*	66,789 (94.8)	8722 (95.0)
*Asian*	1539 (2.2)	264 (2.9)
*Afro-Caribbean*	1188 (1.7)	102 (1.1)
*Other*	930 (1.3)	90 (1.0)
Duration of registration in years, median [IQR]	10.7 [4.6–20.4]	11.0 [3.9–20.9]
Consultation rate in previous year	4 [2–8]	9 [5–15]
**Cardiovascular risk factors**		
Smoking, n (%)		
*Current*	11,276 (12.7)	1443 (16.2)
*Former*	18,343 (20.7)	2287 (25.7)
*Never*	58,919 (66.6)	5186 (58.2)
Diabetes	4955 (4.1)	558 (4.6)
Systolic blood pressure in mmHg, mean (SD)	135 (19.6)	136 (19.5)
Body mass index in kg/m^2^, mean (SD)	26.8 (5.4)	26.7 (5.7)
Total cholesterol in mmol/L, mean (SD)	5.6 (1.1)	5.4 (1.1)
HDL cholesterol in mmol/L, mean (SD)	1.5 (0.4)	1.4 (0.5)
Other autoimmune disease diagnosed, n (%)	6275 (5.2)	1959 (16.1)
**Medication use in previous year**		
DMARDs and biologic therapy, n (%)	125 (0.1)	6172 (50.9)
Nonsteroidal anti-inflammatory drugs, n (%)	10,759 (8.9)	6547 (54.0)
Oral corticosteroids, n (%)	2022 (1.7)	2678 (22.1)
Blood pressure lowering medication, n (%)	27,248 (22.5)	3199 (26.4)
Statins, n (%)	6200 (5.1)	603 (5.0)

Note: DMARDs, disease-modifying anti-rheumatic drugs; HDL, high-density lipoprotein; IQR, interquartile range; RA, rheumatoid arthritis; SD, standard deviation. All patients with rheumatoid arthritis (regardless of disease duration) are included.

*Missing data (%): index of multiple deprivation, 0.3%; ethnic group, 40.3%; BMI, 39.0%; systolic blood pressure, 17.7%; smoking, 26.9%; total cholesterol, 70.7%; HDL cholesterol, 90.5%.

### Association between rheumatoid arthritis and 12 CVDs

**Fig 2:**
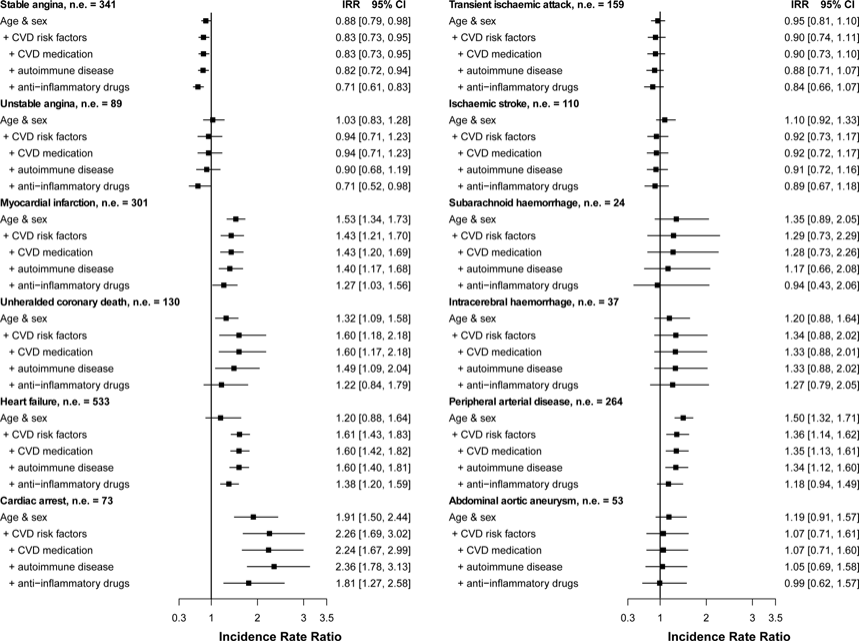
Adjusted incidence rate ratios for the association between rheumatoid arthritis and the initial presentation of twelve cardiovascular diseases. [Fig note: CI, confidence interval; CVD, cardiovascular disease; IRR, incidence rate ratios; n, number of events. CVD risk factors included index of multiple deprivation, smoking status, systolic blood pressure, body mass index and diabetes. All patients with rheumatoid arthritis (regardless of disease duration) are included. Because of the limited number of events, estimates for cardiac arrest, subarachnoid haemorrhage, intracerebral haemorrhage and abdominal aortic aneurysm are adjusted for sex, age, index of multiple deprivation, smoking and diabetes. CVD medications included use of blood pressure and lipid lowering medication. Anti-inflammatory medication included nonsteroidal anti-inflammatory drugs and oral corticosteroids.]

During follow-up, 2,525 (20.8%) RA and 18,146 (15.0%) non-RA individuals experienced a cardiovascular event. The incidence of CVD was higher in patients with RA, 35.33/1000 person-years (95%CI 33.91–36.80) compared with 27.20/1000 person-years (95%CI 26.79–27.62) in non-RA patients for the fatal and non-fatal CVD composite endpoint (Table 2). Individuals with RA had increased incidence rates of AMI, unheralded coronary death, heart failure, cardiac arrest and PAD; and lower rates of stable angina (Fig 2). No evidence of a relationship was found for unstable angina, transient ischaemic attack, ischaemic or haemorrhagic stroke or abdominal aortic aneurysm. Associations were heterogeneous across cardiovascular endpoints (I^2^ = 89.8, p<0.0001). Further adjustment for the presence of other autoimmune diseases and for prescribed CVD and anti-inflammatory medication in the previous year had no effect or tended to decrease the size of associations. Controlling for the effect of specific types of anti-inflammatory drugs generally showed greater reductions in rate ratios for oral corticosteroid use (Figure B in S1 File). However further adjustment for year of entry did not change the estimates (Figure C in S1 File). No evidence of effect modification by sex or age was found for any of the endpoints (Figures D and E in S1 File).

**Table 2:** Incidence of the initial presentation of twelve cardiovascular diseases with 95% confidence interval in individuals with and without rheumatoid arthritis.

	Patients without RA (N = 121,191)		All patients with RA (N = 12,120)		Patients with prevalent RA (N = 6025)		Patients with incident RA (N = 6095)	
	No. of events	Rate / 1000 PY (95% CI)	No. of events	Rate / 1000 PY (95% CI)	No. of events	Rate / 1000 PY (95% CI)	No. of events	Rate / 1000 PY (95% CI)
***Cardiac diseases***								
Stable angina	3455	5.66 (5.48–5.85)	341	5.22 (4.69–5.80)	204	5.55 (4.83–6.36)	137	4.79 (5.48–5.85)
Unstable angina	782	1.28 (1.19–1.37)	89	1.36 (1.11–1.68)	61	1.66 (1.29–1.13)	28	0.98 (0.68–1.42)
Myocardial infarction	1778	2.91 (2.78–3.05)	301	4.61 (4.11–5.16)	203	5.52 (4.81–6.33)	98	3.43 (2.81–4.18)
Unheralded coronary death	887	1.45 (1.36–1.55)	130	1.99 (1.67–2.36)	89	2.42 (1.97–2.98)	41	1.43 (1.06–1.95)
Cardiac arrest	343	0.56 (0.51–0.62)	73	1.12 (0.89–1.40)	46	1.25 (0.94–1.67)	27	0.94 (0.65–1.38)
Heart failure	3006	4.93 (4.75–5.11)	533	8.15 (7.49–8.88)	351	9.54 (8.59–10.59)	182	6.37 (5.51–7.37)
***Cerebrovascular diseases***								
Transient ischaemic attack	1489	2.44 (2.32–2.57)	159	2.43 (2.08–2.84)	98	2.66 (2.19–3.25)	61	2.13 (1.66–2.74)
Ischaemic stroke	885	1.45 (1.36–1.55)	110	1.68 (1.40–2.03)	71	1.93 (1.53–2.44)	39	1.36 (1.00–1.87)
Subarachnoid haemorrhage	163	0.27 (0.23–0.31)	24	0.37 (0.25–0.55)	15	0.41 (0.25–0.68)	9	0.31 (0.16–0.61)
Intracerebral haemorrhage	274	0.45 (0.40–0.51)	37	0.57 (0.41–0.78)	23	0.63 (0.42–0.94)	14	0.49 (0.29–0.83)
***Peripheral vascular diseases***								
Peripheral arterial disease	1569	2.57 (2.45–2.70)	264	4.04 (3.58–4.56)	171	4.65 (4.00–5.40)	93	3.25 (2.66–3.99)
Abdominal aortic aneurysm	395	0.65 (0.59–0.71)	53	0.81 (0.62–1.06)	38	1.03 (0.75–1.42)	15	0.52 (0.32–0.87)
***CVD composite endpoints***								
Coronary & CVD death	8312	13.62 (13.33–13.92)	1030	15.76 (14.83–16.75)	659	17.91 (16.60–19.34)	371	12.98 (11.73–14.37)
Fatal and non-fatal	16,596	27.20 (26.79–27.62)	2309	35.33 (33.91–36.80)	1501	40.80 (38.79–42.92)	808	28.28 (26.39–30.30)

Note: CI, confidence interval; CVD, cardiovascular disease; Non-rheumatoid arthritis estimates are obtained among up to 10 randomly selected patients with no rheumatoid arthritis matched for sex, age, medical practice and index date; PY, person-years of follow-up; RA, rheumatoid arthritis. Rates are unadjusted; Rheumatoid arthritis estimates are obtained among all patients diagnosed with rheumatoid arthritis (regardless of disease duration) who had supporting information for disease diagnosis. The coronary and CVD death composite endpoint includes: stable angina, myocardial infarction, coronary heart diseases not otherwise specified and any cardiovascular death. The fatal and non-fatal CVD composite endpoint additionally includes: non-fatal heart failure, transient ischemic attack, ischemic or haemorrhagic stroke, and peripheral arterial disease.

**Fig 3:**
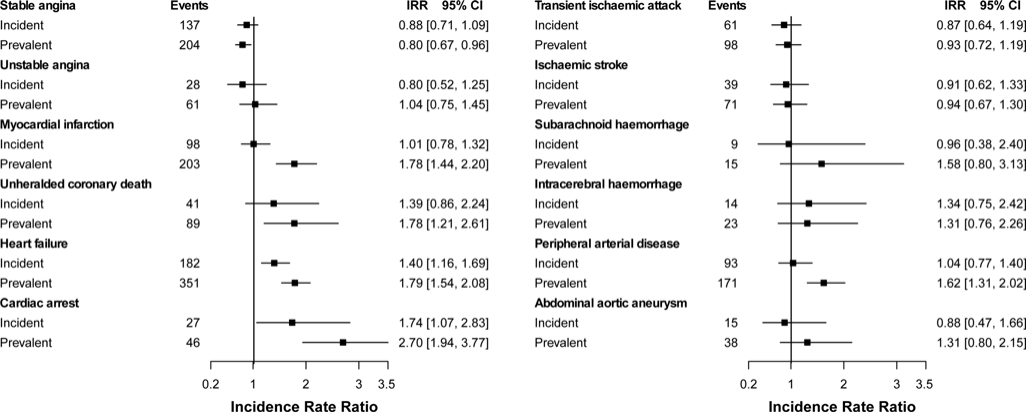
Adjusted incidence rate ratios of the initial presentation of twelve cardiovascular diseases for incident and prevalent rheumatoid arthritis (vs. non-rheumatoid arthritis). [Figure note: CI, confidence interval; IRR, incidence rate ratios adjusted for sex, age, index of multiple deprivation, smoking status, systolic blood pressure, body mass index and diabetes (or rheumatoid arthritis). Because of the limited number of events, estimates for cardiac arrest are not adjusted for body mass index; and estimates for subarachnoid haemorrhage, intracerebral haemorrhage and abdominal aortic aneurysm are adjusted for sex, age, index of multiple deprivation, smoking and diabetes (or rheumatoid arthritis).]

Higher incidence ratios were generally observed for individuals with prevalent than with incident rheumatoid disease compared to those without RA (Fig 3), with stronger evidence for a difference for AMI and PAD. Except for unheralded coronary death, rates of specific CVDs were higher in individuals with recently diagnosed RA (<1 year) than in those with RA disease duration of 2–9 years, but no difference was seen between patients with the shortest (<1 year) and longest (≥10 years) disease duration periods (Figure F in S1 File). In contrast to patients with RA, those with diabetes had also increased rate ratios of stable and unstable angina, ischaemic stroke and intracerebral haemorrhage, but not increased ratios of cardiac arrest (Figure G in S1 File). For statistically significant associations, the size of the estimates was comparable to those of diabetes except for the higher ratios found among diabetic patients (vs. non-diabetic individuals) for stable angina and PAD.

### Results from sensitivity analyses

Estimates of association were robust to sensitivity analyses that excluded individuals diagnosed with another autoimmune disease, excluded individuals with 6 months or more of study follow-up, and censored follow-up two years before the diagnosis of RA in individuals who contributed with study follow-up to both, diseased and non-diseased exposure groups (Table D and Figure H in S1 File). Similarly, associations did not differ when they were compared in the periods before and after the introduction of the pay for performance system in England (April 2004; Figure I in S1 File) or when patients without supporting information for RA diagnosis were also included (Figure J in S1 File). Patterns of associations remained unchanged when first occurrence of CVD was studied regardless of prior presentation with other type of CVD (Figure K in S1 File).

## Discussion

In this comprehensive and contemporary evaluation of the burden of CVDs associated with RA, we reported between thirty-six percent and two-fold higher rates of AMI, unheralded coronary death, heart failure, cardiac arrest and PAD in individuals diagnosed with the disease; but lack of association with unstable angina, cerebrovascular diseases or abdominal aortic aneurysm. Increased rates were found in both individuals with incident and prevalent RA, estimates’ size generally being similar to those observed in diabetic patients. Among individuals with incident RA, the highest CVD rates during follow-up were observed during the first year and after ten years of RA diagnosis. Rates were similar in men and women and findings were robust to multiple sensitivity analyses.

The strengths of this analysis are the population-based design of broad age span (18–99 years), large sample size (N = 133,311 in the main analysis), a cohort including all ranges of RA severity and information from individuals treated in recent years (1997–2010), and the study of the twelve most common clinical presentations of CVDs. Previous studies assessed only a limited range of cardiovascular endpoints, generally myocardial infarction and all-cause stroke, and most were small or were undertaken prior to the widespread use of modern therapies for rheumatoid arthritis and to the introduction of an earlier (i.e. within 3 months of onset) and more aggressive pharmacological management of patients with a combination of DMARDs and with short-courses of glucocorticoids for the management of flares. The cohort design and the unique data source linking EHRs covering primary care, hospital and mortality data allowed identification of a non-RA group selected from the same population and improved ascertainment of cardiovascular outcomes, which have been found to be valid[31,32] and were determined independently of RA status. Unlike previous studies, we were able to study not only the effect of adjusting for the presence of established cardiovascular risk factors but also for prescribed anti-inflammatory and cardiovascular medication currently used in clinical practice and for the presence of other diagnosed autoimmune diseases. We were also able to investigate in the same study differences in associations between patients with prevalent and incident RA, and according to RA disease duration, providing the most comprehensive picture of the burden of CVDs related to RA published to date.

One of the limitations of the study, inherent to the use of EHRs, was the possibility of RA diagnosis misclassification (e.g. mild or atypical RA patients might be less likely to be diagnosed and some diagnoses made in primary care could be inaccurate). However, we used the same diagnosis codes previously validated within the same or analogous medical databases and for which positive predictive values between 74% and 81% have been reported[27,28], and the primary analysis included only RA patients with supporting information for diagnosis. Typical DMARD medication or record of multiple diagnosis codes in primary care have been shown to increase the validity of RA diagnosis by general practitioners (≥80% sensitivity and specificity)[33]. Furthermore, although changes in clinical diagnosis and management over time could affect the estimates, ensuring that non-disease matched individuals were registered in the same practices at study inclusion minimised the introduction of this type of bias. Model adjustment for calendar-year had also negligible effects on the estimates (Figure C in S1 File).

In the cohort analysed the distribution of established cardiovascular risk factors was similar in individuals with or without RA, except for the higher prevalence of current or former smoking among RA patients. Smoking has been identified as a risk factor for the development of RA in previous studies[17] and for high antibody titres of rheumatoid factor and anti-citrullinated protein[18]. In our analysis, adjustment for the effect of smoking and other cardiovascular risk factors measured at baseline decreased the size of the estimates but did not completely explain the observed increased risk of CVDs in RA patients. However, residual confounding resulting from lack of information on known (e.g. rheumatoid factor status or lipids) or unknown risk factors cannot be completely excluded. Furthermore, precise information about time of diagnosis and the duration of and compliance with medication was not available and changes in medication use over time associated with RA flares were not investigated. The effect of anti-inflammatory drugs on CVD is complex. While reductions of cardiovascular risk could be mediated through their anti-inflammatory properties, they could also facilitate progression of atherosclerosis (e.g. corticosteroid could increase cardiovascular risk through effects on blood pressure, lipid profile and insulin resistance[34]). Indeed a meta-analysis of 280 trials of nonsteroidal anti-inflammatory drugs showed increased risk of coronary heart disease and death and heart failure associated with coxibs, diclofenac or ibuprofen use[35]. Alternatively, because oral corticosteroids and nonsteroidal anti-inflammatory drugs are generally prescribed when substantial joint inflammation is present, adjusting for anti-inflammatory medication could result in estimate underestimation. In our study, additionally adjusting for baseline prescription of anti-inflammatory medication further decreased the magnitude of the estimates but associations with myocardial infarction, heart failure and cardiac arrest remained statistically significant.

The duration of follow-up was limited, with a median time of four years per patient, but 2,125 patients with incident RA contributed with ten years or more of study follow-up to the analysis. The increased risk of CVD was observed at both, early stages of the disease and ten years or more after disease diagnosis, and in both patients with incident and prevalent disease. Results were robust to exclusion of patients who received oral corticosteroids and DMARDs or biologic therapy in the year before study entry. Although it is debated whether the timing of increase in risk of CVD is before or soon after the clinical onset of RA[18], our findings are consistent with smaller longitudinal studies reporting higher risk of coronary heart disease[36,37] and congestive heart failure[38] early after disease diagnosis. Our results suggest that the mechanism by which RA increases the cardiovascular risk is not exclusively related to a cumulative inflammatory burden but to other shared pathogenic mechanisms between the two types of disease[38].

The observed associations are consistent with results from meta-analyses that reported increased risk of fatal[6] and non-fatal[39] AMI, absence of relationship with stroke[3] and of sex-differences in these associations[39]. The lower risk of stable angina and the increased risk of initial presentation of CVD with unheralded coronary death in patients with RA are of clinical relevance and consistent with reports of a higher risk of undiagnosed mild vascular disease and unrecognised AMI and sudden cardiac death[40] in RA patients who suffer from chronic pain and frequently use analgesic drugs[18,40]. Although other less common mechanisms cannot be excluded (e.g. sensory disorders associated with neurological complications), these findings suggest that some deaths could be averted if patients are adequately informed of the high risk of unrecognised coronary disease and educated on how to recognise the symptoms. In our study, we did not find evidence of association between RA status and different types of cerebrovascular diseases, including ischaemic and haemorrhagic stroke. Evidence of the association between stroke and RA is conflicting and while a review reported increased risk of AMI but not stroke^3^, several meta-analyses found increased risk of both fatal and non-fatal endpoints[4,6,39]. Because higher risk of stroke was often reported in large studies and inception cohorts usually failed to find evidence of association, suggested reasons for discrepancies have been a shorter duration of RA disease and/or follow-up, less disease severity, differences in clinical management of patients (i.e. more aggressive in recent years) or age distributions in RA populations, lack of power, or absence of adjustment for the effect of cardiovascular risk factors. The numbers of events for some of the subtypes of stroke limited the power of some of the analyses but, in contrast to estimations for diabetes, we did not find evidence of association not only between RA (all RA patients, or patients with prevalent or incident RA) and specific types of stroke but also with a composite endpoint of all types of stroke investigated (data not shown) in a population with no prior history of CVD. Future analyses in larger populations and/or when longer follow-up data will be available will help to understand better the reasons for the observed lack of association with cerebrovascular endpoints.

The magnitude of cardiovascular risk estimates (similar to diabetes for several CVDs) has implications for clinical practice and supports the incorporation of regular determination and monitoring of cardiovascular risk early after RA diagnosis. While current ERC[41] and EULAR[42] guidelines recognise the importance of adequate clinical management of patients with RA to prevent CVDs, the equivalent USA guidelines, including new ACC/AHA recommendations[43], do not mention the need for cardiovascular assessment and management. Furthermore, although no intervention studies have been conducted to evaluate the effectiveness of implementing primary prevention therapy and monitoring of targets for RA patients, the study findings support the need for this type of research. Likewise, evidence of the association between RA and subsequent development of specific cardiovascular conditions other than AMI (e.g. heart failure and cardiac arrest) but a lack of relationship with others (e.g. cerebrovascular disease) in the general population has important implications for risk prediction, given that current recommended risk scores for patients with RA have been developed using angina, AMI and cerebrovascular endpoints[44].

In conclusion, regardless of the mechanisms for the observed associations with some but not all types of CVDs, our findings are important to inform both clinical practice and the choice of cardiovascular endpoints for risk prediction model development and trials targeted at patients with RA.

## Supporting Information

S1 File Supplemental Methods: Multiple imputation. **Tables:** Table A. Read and ICD 10 diagnosis codes for rheumatoid arthritis. Table B. List of drugs included as disease-modifying anti-rheumatic drugs and biologic therapy. Table C. List of diseases considered in the ‘other autoimmune disease’ covariate. Table D. Adjusted incidence rate ratios for the association between rheumatoid arthritis and the initial presentation of cardiovascular diseases (composite endpoints). Table E. Adjusted incidence rate ratios for the association between anti-inflammatory medication use and twelve cardiovascular diseases. **Figures:** Figure A. Electronic health record phenotyping algorithm for rheumatoid arthritis. Figure B. Adjusted incidence rate ratios for the association between rheumatoid arthritis and the initial presentation of twelve cardiovascular diseases, additionally adjusted for the effect of anti-inflammatory medication. Figure C. Adjusted incidence rate ratios for the association between rheumatoid arthritis and the initial presentation of twelve cardiovascular diseases further adjusted of year of entry. Figure D. Adjusted incidence rate ratios for the association between rheumatoid arthritis and the initial presentation of twelve cardiovascular diseases in men and women. Figure E. Adjusted incidence rate ratios for the association between rheumatoid arthritis and the initial presentation of twelve cardiovascular diseases stratified by age group. Figure F. Adjusted incidence rate ratios for the initial presentation of cardiovascular disease stratified by disease duration among patients with rheumatoid arthritis. Figure G. Adjusted incidence rate ratios of the initial presentation of twelve cardiovascular diseases for all patients with rheumatoid arthritis (vs. non-rheumatoid arthritis), and for all patients with diabetes (vs. non-diabetes). Figure H. Adjusted incidence rate ratios for the association between rheumatoid arthritis and the initial presentation of twelve cardiovascular diseases: results from sensitivity analyses. Figure I. Adjusted incidence rate ratios for the association between rheumatoid arthritis and the initial presentation of twelve cardiovascular diseases before and after the introduction of pay for performance (April 2004). Figure J. Comparison of adjusted incidence rate ratios for the association between rheumatoid arthritis and the initial presentation of twelve cardiovascular diseases according to disease definition. Figure K. Adjusted incidence rate ratios for the association between rheumatoid arthritis and the initial and first presentation of twelve cardiovascular diseases.
(DOCX)
