# Supplementary material for: Rheumatoid Arthritis and Incidence of Twelve Initial Presentations of Cardiovascular Disease: A Population Record-Linkage Cohort Study in England
Source: PLoS One. 2016 Mar 15;11(3):e0151245. doi: 10.1371/journal.pone.0151245 (PMC4792375; doi:10.1371/journal.pone.0151245)
Supplement: S1 File — Tables: Table A. Read and ICD 10 diagnosis codes for rheumatoid arthritis. Table B. List of drugs included as disease-modifying anti-rheumatic drugs and biologic therapy. Table C. List of diseases considered in the ‘other autoimmune disease’ covariate. Table D. Adjusted incidence rate ratios for the association between rheumatoid arthritis and the initial presentation of cardiovascular diseases (composite endpoints). Table E. Adjusted incidence rate ratios for the association between anti-inflammatory medication use and twelve cardiovascular diseases. Figures: Figure A. Electronic health record phenotyping algorithm for rheumatoid arthritis. Figure B. Adjusted incidence rate ratios for the association between rheumatoid arthritis and the initial presentation of twelve cardiovascular diseases, additionally adjusted for the effect of anti-inflammatory medication. Figure C. Adjusted incidence rate ratios for the association between rheumatoid arthritis and the initial presentation of twelve cardiovascular diseases further adjusted of year of entry. Figure D. Adjusted incidence rate ratios for the association between rheumatoid arthritis and the initial presentation of twelve cardiovascular diseases in men and women. Figure E. Adjusted incidence rate ratios for the association between rheumatoid arthritis and the initial presentation of twelve cardiovascular diseases stratified by age group. Figure F. Adjusted incidence rate ratios for the initial presentation of cardiovascular disease stratified by disease duration among patients with rheumatoid arthritis. Figure G. Adjusted incidence rate ratios of the initial presentation of twelve cardiovascular diseases for all patients with rheumatoid arthritis (vs. non-rheumatoid arthritis), and for all patients with diabetes (vs. non-diabetes). Figure H. Adjusted incidence rate ratios for the association between rheumatoid arthritis and the initial presentation of twelve cardiovascular diseases: results from sensitivity analyses [file pone.0151245.s001.docx]

# SUPPLEMENTAL METHODS

## Multiple imputation

Risk factor data appeared to be missing at random after adjusting for major confounders (e.g. age, sex, diabetes, BMI and blood pressure). Hence, multiple imputation was implemented using the *M*^1^ algorithm in the statistical package Stata 13.1, to replace missing values in exposure and risk factor variables. Imputation models were estimated separately for men and women and included:

1. all the baseline covariates used in the main analysis (age, quadratic age, diabetes, smoking, systolic blood pressure, total cholesterol, HDL cholesterol, index of multiple deprivation; diagnosis of another autoimmune disease);
2. prior (between 1 and 4 years before study entry) and post (between 0 and 1 year after study entry) averages of continuous covariates in the main analysis;
3. baseline, prior and post average measurements of covariates not considered in the main analysis (diastolic blood pressure, alcohol intake, white blood cell count, haemoglobin, creatinine);
4. rheumatoid arthritis status (no, incident, prevalent);
5. positive rheumatoid factor on or prior to the date of entry;
6. baseline medications (nonsteroidal anti-inflammatory drugs, disease-modifying anti-rheumatic drugs / biologics, oral corticosteroids, antiplatelet medication, statins, blood pressure lowering medication, low-dose aspirin, loop diuretics, oral contraceptives and hormone replacement therapy);
7. coexisting medical conditions (hypertension, history of depression, cancer, renal disease, liver disease and chronic obstructive pulmonary disease);
8. the Nelson-Aalen hazard and the event status for each of the 12 endpoints analysed^2^.

Non-normally distributed variables were log-transformed for imputation and back-exponentiated to their original scale for analysis. Five multiply imputed datasets were generated, and Poisson models were fitted to each dataset. Coefficients were combined using Rubin’s rules. The Kolmogorov-Smirnov test was used to compare the distribution of observed versus imputed log-transformed covariates.

**Reference list**

1. Royston P, White IR, Multiple imputation by chained equations (MICE): Implementations in STATA, Journal of Statistical Software, 2011; **45**(4): 1-20.
2. van Buuren S. Multiple imputation of discrete and continuous data by fully conditional specification. Stat Methods Med Res. 2007; **16**: 219-242.

# SUPPLEMENTAL TABLES

## Table A. Read and ICD 10 diagnosis codes for rheumatoid arthritis

| **Read codes** | |
| --- | --- |
| 2G27.00 | O/E-hands-rheumatoid spindling |
| 66H..13 | Rheumatoid arthritis monitoring |
| F371200 | Polyneuropathy in rheumatoid arthritis |
| F396400 | Myopathy due to rheumatoid arthritis |
| G5y8.00 | Rheumatoid myocarditis |
| G5yA.00 | Rheumatoid carditis |
| H570.00 | Rheumatoid lung |
| N005.00 | Adult Still's Disease |
| N040.00 | Rheumatoid arthritis |
| N040000 | Rheumatoid arthritis of cervical spine |
| N040100 | Other rheumatoid arthritis of spine |
| N040200 | Rheumatoid arthritis of shoulder |
| N040500 | Rheumatoid arthritis of elbow |
| N040600 | Rheumatoid arthritis of distal radio-ulnar joint |
| N040700 | Rheumatoid arthritis of wrist |
| N040800 | Rheumatoid arthritis of MCP joint |
| N040900 | Rheumatoid arthritis of PIP joint of finger |
| N040A00 | Rheumatoid arthritis of DIP joint of finger |
| N040B00 | Rheumatoid arthritis of hip |
| N040D00 | Rheumatoid arthritis of knee |
| N040F00 | Rheumatoid arthritis of ankle |
| N040G00 | Rheumatoid arthritis of subtalar joint |
| N040H00 | Rheumatoid arthritis of talonavicular joint |
| N040J00 | Rheumatoid arthritis of other tarsal joint |
| N040K00 | Rheumatoid arthritis of 1st MTP joint |
| N040N00 | Rheumatoid vasculitis |
| N040P00 | Seronegative rheumatoid arthritis |
| N040Q00 | Rheumatoid bursitis |
| N040R00 | Rheumatoid nodule |
| N040S00 | Rheumatoid arthritis - multiple joint |
| N040T00 | Flare of rheumatoid arthritis |
| N041.00 | Felty's syndrome |
| N042.00 | Other rheumatoid arthropathy + visceral/systemic involvement |
| N042100 | Rheumatoid lung disease |
| N042200 | Rheumatoid nodule |
| N042z00 | Rheumatoid arthropathy + visceral/systemic involvement NOS |
| N047.00 | Seropositive errosive rheumatoid arthritis |
| N04X.00 | Seropositive rheumatoid arthritis, unspecified |
| N04y000 | Rheumatoid lung |
| N04y011 | Caplan's syndrome |
| N04y012 | Fibrosing alveolitis associated with rheumatoid arthritis |
| N04y200 | Adult-onset Still's disease |
| Nyu1100 | [X]Other seropositive rheumatoid arthritis |
| Nyu1200 | [X]Other specified rheumatoid arthritis |
| Nyu1G00 | [X]Seropositive rheumatoid arthritis, unspecified |
| N040300 | Rheumatoid arthritis of sternoclavicular joint |
| N040400 | Rheumatoid arthritis of acromioclavicular joint |
| N040C00 | Rheumatoid arthritis of sacro-iliac joint |
| N040E00 | Rheumatoid arthritis of tibio-fibular joint |
| N040L00 | Rheumatoid arthritis of lesser MTP joint |
| N040M00 | Rheumatoid arthritis of IP joint of toe |
| Nyu1000 | [X]Rheumatoid arthritis+involvement/other organs or systems |
|  |  |
| **ICD 10 codes** | |
| I528 | Rheumatoid carditis |
| J990 | Rheumatoid lung disease |
| M05 | Seropositive rheumatoid arthritis |
| M050 | Felty's syndrome |
| M051 | Rheumatoid lung disease |
| M052 | Rheumatoid vasculitis |
| M053 | Rheumatoid arthritis with involvement of other organs and sys |
| M058 | Other seropositive rheumatoid arthritis |
| M059 | Seropositive rheumatoid arthritis, unspecified |
| M06 | Other rheumatoid arthritis |
| M060 | Seronegative rheumatoid arthritis |
| M061 | Adult-onset Still's disease |
| M062 | Rheumatoid bursitis |
| M063 | Rheumatoid nodule |
| M068 | Other specified rheumatoid arthritis |
| M069 | Rheumatoid arthritis, unspecified |

## Table B. List of drugs included as disease-modifying anti-rheumatic drugs and biologic therapy suggestive of rheumatoid arthritis

Adalimumab

Anakinra

Azathioprine

Aurothiomalate, auranofin

Certolizumab pegol

Chloroquine / hydroxychloroquine

Cyclosporin

Cyclophosphamide

Etanercept

Golimumab

Infliximab

Leflunomide

Mepacrine hydrochloride

Methotrexate

Penicillamine

Rituximab

Sulfasalazine

Tocilizumab

## Table C. List of diseases considered in the “other autoimmune disease” covariate

Ankylosing spondylitis

Autoimmune bullous disease

Autoimmune uveitis

Behcet’s disease

Dermato-polymyositis

Giant cell arteritis

Henoch-Schönlein purpura

Multiple sclerosis

Polyarteritis nodosa

Polymyalgia rheumatica

Primary biliary cirrhosis

Psoriasis

Sjögren’s syndrome

Systemic lupus erythematosus

Systemic sclerosis

Wegener’s granulomatosis

## Table D. Adjusted incidence rate ratios for the association between rheumatoid arthritis and the initial presentation of cardiovascular diseases (composite endpoints)

|  | **Coronary and CVD death composite** | | |  | **Fatal and non-fatal CVD composite** | | |
| --- | --- | --- | --- | --- | --- | --- | --- |
|  | **All patients with RA** | **Patients with incident RA** | **Patients with prevalent RA** |  | **All patients with RA** | **Patients with incident RA** | **Patients with prevalent RA** |
|  | **IRR (95%CI)** | **IRR (95%CI)** | **IRR (95%CI)** |  | **IRR (95%CI)** | **IRR (95%CI)** | **IRR (95%CI)** |
| Adjusted for age and sex | 1.11 (1.04-1.18) | 0.94 (0.85-1.05) | 1.23 (1.13-1.33) |  | 1.21 (1.17-1.26) | 1.04 (0.98-1.10) | 1.36 (1.30-1.43) |
| + CVD risk factors | 1.02 (0.93-1.11) | 0.93 (0.82-1.07) | 1.08 (0.97-1.21) |  | 1.16 (1.09-1.23) | 1.03 (0.94-1.13) | 1.26 (1.18-1.35) |
| + other autoimmune disease | 1.00 (0.92-1.10) | 0.92 (0.80-1.05) | 1.07 (0.96-1.20) |  | 1.14 (1.08-1.21) | 1.01 (0.92-1.11) | 1.25 (1.17-1.33) |
| + year of entry | 1.01 (0.92-1.11) | 1.03 (0.90-1.17) | 1.00 (0.89-1.12) |  | 1.15 (1.09-1.22) | 1.13 (1.03-1.24) | 1.17 (1.09-1.26) |
| + Exclusion of patients |  |  |  |  |  |  |  |
| with other autoimmune disease | 1.02 (0.93-1.13) | 0.94 (0.81-1.08) | 1.09 (0.96-1.23) |  | 1.16 (1.09-1.24) | 1.03 (0.93-1.13) | 1.26 (1.17-1.36) |
| ≤6 months of follow-up | 1.00 (0.91-1.10) | 0.88 (0.76-1.01) | 1.10 (0.98-1.24) |  | 1.14 (1.07-1.21) | 0.97 (0.88-1.06) | 1.28 (1.19-1.37) |
| + Exclusion of 2yr unexposed follow-up for mixed cases^*^ | 1.01 (0.93-1.11) | 0.93 (0.82-1.06) | 1.08 (0.96-1.21) |  | 1.15 (1.09-1.22) | 1.02 (0.93-1.12) | 1.26 (1.18-1.34) |
| + Inclusion of patients with & without SID | 1.04 (0.96-1.12) | 0.98 (0.87-1.11) | 1.09 (0.99-1.20) |  | 1.16 (1.11-1.22) | 1.04 (0.97-1.13) | 1.27 (1.20-1.35) |
| + Inclusion of first presentation of CVD^**^ | 1.09 (1.01-1.17) | 0.93 (0.82-1.07) | 1.08 (0.97-1.21) |  | 1.14 (1.08-1.21) | 1.03 (0.94-1.13) | 1.26 (1.18-1.35) |
|  |  |  |  |  |  |  |  |

Note: CI, confidence intervals; CVD, cardiovascular disease; IRR, incidence rate ratios; RA, rheumatoid arthritis; SID, supportive information of disease. Cardiovascular risk factors included index of multiple deprivation, smoking status, systolic blood pressure, body mass index and diabetes. The coronary and CVD death composite endpoint includes: stable angina, myocardial infarction, coronary heart diseases not otherwise specified and any cardiovascular death. The fatal and non-fatal CVD composite endpoint additionally includes: non-fatal heart failure, transient ischemic attack, ischemic or haemorrhagic stroke, and peripheral arterial disease.

* Censoring of follow-up two years before diagnosis of rheumatoid arthritis for patients who contributed to rheumatoid and non-rheumatoid analysis groups (i.e. patients with incident disease).

** Endpoints were the first presentation of the specific cardiovascular disease type regardless of prior occurrence of another type of cardiovascular disease.

## Table E. Adjusted incidence rate ratios for the association between anti-inflammatory medication use and the initial presentation of twelve cardiovascular diseases

|  | **NSAIDS** | |  | **Oral corticosteroids** | |
| --- | --- | --- | --- | --- | --- |
|  | **All patients**  **IRR (95%CI)** | **RA patients**  **IRR (95%CI)** |  | **All patients**  **IRR (95%CI)** | **RA patients**  **IRR (95%CI)** |
| ***Cardiac diseases*** |  |  |  |  |  |
| Stable angina | 1.26 (1.12-1.41) | 0.98 (0.78-1.24) |  | 1.24 (1.02-1.52) | 1.29 (0.94-1.78) |
| Unstable angina | 1.40 (1.12-1.75) | 1.29 (0.77-2.17) |  | 1.56 (1.07-2.28) | 1.35 (0.75-2.41) |
| Myocardial infarction | 1.11 (0.95-1.28) | 1.03 (0.78-1.37) |  | 1.34 (1.05-1.71) | 1.10 (0.81-1.49) |
| Unheralded coronary death | 1.20 (0.88-1.63) | 0.99 (0.62-1.59) |  | 1.85 (1.27-2.70) | 2.04 (1.10-3.79) |
| Heart failure | 1.04 (0.93-1.16) | 0.95 (0.78-1.16) |  | 1.60 (1.35-1.90) | 1.43 (1.12-1.81) |
| Cardiac arrest | 1.14 (0.83-1.56) | 1.98 (1.09-3.60) |  | 1.78 (1.22-2.59) | 2.09 (1.22-3.57) |
| ***Cerebrovascular diseases*** |  |  |  |  |  |
| Transient ischaemic attack | 1.00 (0.82-1.21) | 0.90 (0.59-1.36) |  | 1.31 (0.98-1.76) | 1.21 (0.73-2.00) |
| Ischaemic stroke | 1.10 (0.87-1.40) | 0.65 (0.41-1.04) |  | 1.00 (0.66-1.50) | 1.00 (0.57-1.77) |
| Subarachnoid haemorrhage | 1.08 (0.60-1.94) | - |  | 2.71 (1.23-5.99) | - |
| Intracerebral haemorrhage | 0.97 (0.65-1.44) | 0.82 (0.37-1.77) |  | 1.26 (0.69-2.31) | 0.85 (0.34-2.13) |
| ***Peripheral vascular diseases*** |  |  |  |  |  |
| Peripheral arterial disease | 1.01 (0.82-1.23) | 0.70 (0.50-0.97) |  | 1.61 (1.22-2.12) | 1.49 (1.01-2.21) |
| Abdominal aortic aneurysm | 0.74 (0.49-1.10) | 0.81 (0.37-1.76) |  | 1.96 (1.20-3.18) | 2.22 (1.01-4.90) |
| ***CVD composite endpoints*** |  |  |  |  |  |
| Coronary and CVD death | 1.18 (1.09-1.27) | 1.02 (0.88-1.18) |  | 1.32 (1.16-1.50) | 1.34 (1.12-1.61) |
| Fatal and non-fatal CVD | 1.10 (1.04-1.16) | 0.95 (0.86-1.06) |  | 1.37 (1.26-1.49) | 1.31 (1.16-1.48) |

Note: CI, confidence interval; NSAIDS, nonsteroidal anti-inflammatory drugs; IRR, incidence rate ratios were adjusted for sex, age, index of multiple deprivation, smoking status, systolic blood pressure, body mass index, diabetes, and additionally for rheumatoid arthritis status in the analysis including all patients; RA, rheumatoid arthritis. The coronary and CVD death composite endpoint includes: stable angina, myocardial infarction, coronary heart diseases not otherwise specified and any cardiovascular death. The fatal and non-fatal CVD composite endpoint additionally includes: non-fatal heart failure, transient ischemic attack, ischemic or haemorrhagic stroke, and peripheral arterial disease. Because of the limited number of events estimates for cardiac arrest are not adjusted for body mass index; and estimates for subarachnoid haemorrhage, intracerebral haemorrhage and abdominal aortic aneurysm are adjusted for sex, age, index of multiple deprivation, smoking and diabetes. No estimates were computed for subarachnoid haemorrhage within the group of rheumatoid arthritis patients because of the small number of events recorded during follow-up.

# SUPPLEMENTAL FIGURES

## Figure A. Electronic health record phenotyping algorithm for rheumatoid arthritis


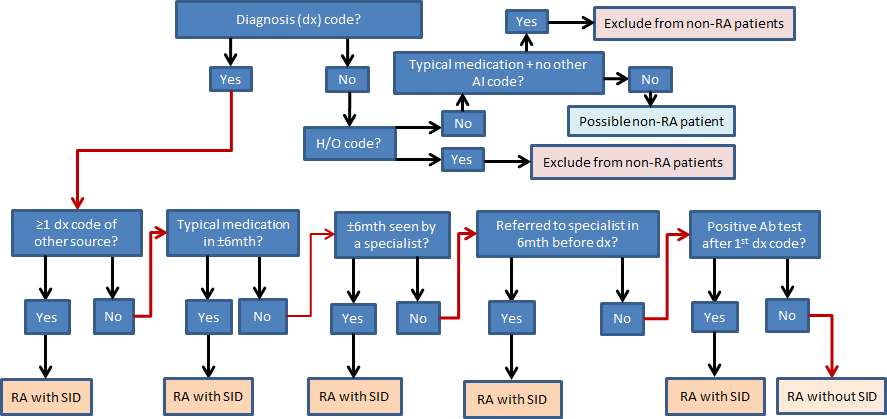


Note: Ab test, antibody test (i.e. rheumatoid factor and/or anti-cyclic citrullinated peptide antibody test); AI, autoimmune disease; dx code, Read diagnosis code from the Clinical Practice Research Datalink (CPRD, primary care) or ICD 10 code from the Hospital Episode Statistics (HES, hospital); H/O, history of; RA, rheumatoid arthritis; SID, supportive information for rheumatoid arthritis diagnosis; typical medication, disease-modifying anti-rheumatic drug or biologic therapy. Primary analyses only included RA patients with supportive information for diagnosis. In sensitivity analyses RA patients with and without supportive information were included. Patients with juvenile rheumatoid arthritis were excluded.

## Figure B. Adjusted incidence rate ratios for the association between rheumatoid arthritis and the initial presentation of twelve cardiovascular diseases, additionally adjusted for the effect of anti-inflammatory medication

**

**

Note: CI, confidence interval; CVD, cardiovascular disease; IRR, incidence rate ratios; NSAIDS, nonsteroidal anti-inflammatory drugs; n, number of events. Cardiovascular disease risk factors include index of multiple deprivation, smoking status, systolic blood pressure, body mass index and diabetes. Rheumatoid arthritis estimates are obtained among all patients diagnosed with rheumatoid arthritis (regardless of disease duration) who had supporting information for disease diagnosis. Because of the limited number of events estimates for cardiac arrest, subarachnoid haemorrhage, intracerebral haemorrhage and abdominal aortic aneurysm are adjusted index of multiple deprivation, smoking and diabetes.

## Figure C. Adjusted incidence rate ratios for the association between rheumatoid arthritis and the initial presentation of twelve cardiovascular diseases further adjusted for year of entry





Note: CI, confidence interval; IRR, incidence rate ratios adjusted for sex, age, year of study entry, index of multiple deprivation, smoking status, systolic blood pressure, body mass index, diabetes and year of study entry. Rheumatoid arthritis estimates are obtained among all patients diagnosed with rheumatoid arthritis (regardless of disease duration) who had supporting information for disease diagnosis. Because of the limited number of events estimates for cardiac arrest, subarachnoid haemorrhage, intracerebral haemorrhage and abdominal aortic aneurysm are adjusted for sex, age, year of study entry, index of multiple deprivation, smoking and diabetes.

## Figure D. Adjusted incidence rate ratios for the association between rheumatoid arthritis and the initial presentation of twelve cardiovascular diseases in men and women





Note: CI, confidence interval; IRR, incidence rate ratios adjusted for age, index of multiple deprivation, smoking status, systolic blood pressure, body mass index and diabetes. Rheumatoid arthritis estimates are obtained among all patients diagnosed with rheumatoid arthritis (regardless of disease duration) who had supporting information for disease diagnosis. All subarachnoid haemorrhage events occurred in women. Because of the limited number of events estimates for cardiac arrest, intracerebral haemorrhage and abdominal aortic aneurysm are adjusted for sex, index of multiple deprivation, smoking and diabetes.

## Figure E. Adjusted incidence rate ratios for the association between rheumatoid arthritis and the initial presentation of twelve cardiovascular diseases stratified by age group





Note: CI, confidence interval; IRR, incidence rate ratios adjusted for sex, index of multiple deprivation, smoking status, systolic blood pressure, body mass index and diabetes. Rheumatoid arthritis estimates are obtained among all patients diagnosed with rheumatoid arthritis (regardless of disease duration) who had supporting information for disease diagnosis. Because of the limited number estimates for cardiac arrest, subarachnoid haemorrhage, intracerebral haemorrhage and abdominal aortic aneurysm are adjusted for sex, index of multiple deprivation and diabetes.

## Figure F. Adjusted incidence rate ratios for the initial presentation of cardiovascular disease by disease duration (vs. <1 year duration) among patients with rheumatoid arthritis

**

**

Note: CI, confidence interval; IRR, incidence rate ratios adjusted for sex, age, index of multiple deprivation, smoking status, systolic blood pressure, body mass index and diabetes. Estimations for subarachnoid haemorrhage and abdominal aortic aneurysm were not computed because of the small number of events. In addition, because of the limited number of events estimates for cardiac arrest are not adjusted for body mass index.

## Figure G. Adjusted incidence rate ratios of the initial presentation of twelve cardiovascular diseases for all patients with rheumatoid arthritis (vs. non-rheumatoid arthritis), and for all patients with diabetes (vs. non-diabetes)

##



Note: CI, confidence interval; IRR, incidence rate ratios adjusted for sex, index of multiple deprivation, smoking status, systolic blood pressure, body mass index and diabetes. Rheumatoid arthritis estimates are obtained among all patients diagnosed with rheumatoid arthritis (regardless of disease duration) who had supporting information for disease diagnosis. Because of the limited number estimates for cardiac arrest, subarachnoid haemorrhage, intracerebral haemorrhage and abdominal aortic aneurysm are adjusted for sex, index of multiple deprivation and diabetes.

## Figure H. Adjusted incidence rate ratios for the association between rheumatoid arthritis and the initial presentation of twelve cardiovascular diseases: results from sensitivity analyses





Note: CI, confidence interval; CVD, cardiovascular disease; IRR, incidence rate ratios adjusted for sex, age, index of multiple deprivation, smoking status, systolic blood pressure, body mass index and diabetes. Rheumatoid arthritis estimates are obtained among all patients diagnosed with rheumatoid arthritis (regardless of disease duration) who had supporting information for disease diagnosis. Because of the limited number of events estimates for cardiac, subarachnoid haemorrhage, intracerebral haemorrhage and abdominal aortic aneurysm are adjusted for sex, age, index of multiple deprivation, smoking and diabetes. Sensitivity analyses were:

**Second row:** Exclusion of patients with a diagnosis of another autoimmune disorder.

**Third row:** Exclusion of patients with less than 6 months of study follow-up.

**Fourth row:** Exclusion of 2 years of study follow-up before the diagnosis of an incident rheumatoid arthritis for patients who contributed with follow-up to the rheumatoid arthritis and non-rheumatoid arthritis groups.

## Figure I. Adjusted incidence rate ratios for the association between rheumatoid arthritis and the initial presentation of twelve cardiovascular diseases before and after the introduction of pay for performance (April 2004)





Note: CI, confidence interval; IRR, incidence rate ratios adjusted for sex, age, index of multiple deprivation, smoking status, systolic blood pressure, body mass index and diabetes; QoF, Quality of Outcomes Framework. Rheumatoid arthritis estimates are obtained among all patients diagnosed with rheumatoid arthritis (regardless of disease duration) who had supporting information for disease diagnosis. Because of the limited number of events estimates for cardiac arrest, subarachnoid haemorrhage, intracerebral haemorrhage and abdominal aortic aneurysm are adjusted for sex, age, index of multiple deprivation, smoking and diabetes.

## Figure J. Comparison of adjusted incidence rate ratios for the association between rheumatoid arthritis and the initial presentation of twelve cardiovascular diseases according to disease definition





Note: CI, confidence interval; IRR, incidence rate ratios adjusted for sex, age, index of multiple deprivation, smoking status, systolic blood pressure, body mass index and diabetes. Because of the limited number of events estimates for cardiac arrest, subarachnoid haemorrhage, intracerebral haemorrhage and abdominal aortic aneurysm are adjusted for sex, age, index of multiple deprivation, smoking and diabetes. SID, supportive information for rheumatoid arthritis diagnosis. The population with SID included all patients with rheumatoid arthritis (regardless of disease duration) who had supportive information of diagnosis and the corresponding up to 10 patients without rheumatoid arthritis matched for age, sex, medical practice and index date.

## Figure K. Adjusted incidence rate ratios for the association between rheumatoid arthritis and the initial and first presentation of twelve cardiovascular diseases





Note: CI, confidence interval; CVD, cardiovascular disease; IRR, incidence rate ratios adjusted for sex, age, index of multiple deprivation, smoking status, systolic blood pressure, body mass index and diabetes. Rheumatoid arthritis estimates are obtained among all patients diagnosed with rheumatoid arthritis (regardless of disease duration) who had supporting information for disease diagnosis. Because of the limited number of events estimates for cardiac arrest, subarachnoid haemorrhage, intracerebral haemorrhage and abdominal aortic aneurysm are adjusted for sex, age, index of multiple deprivation, smoking and diabetes. The initial CVD presentation was the first presentation of any cardiovascular disease experienced by a patient (e.g. a myocardial infarction for a patient who experienced first a myocardial infarction and later developed heart failure). The first event of this type was the first presentation of cardiovascular disease for a patient regardless of prior occurrence of another type of cardiovascular disease (e.g. a patient who experienced first a myocardial infarction and later heart failure would contribute to both myocardial infarction and heart failure estimations).
